# Light manipulation of nanoparticles in arrays of topological defects

**DOI:** 10.1038/srep20742

**Published:** 2016-02-17

**Authors:** D. Kasyanyuk, P. Pagliusi, A. Mazzulla, V. Reshetnyak, Yu. Reznikov, C. Provenzano, M. Giocondo, M. Vasnetsov, O. Yaroshchuk, G. Cipparrone

**Affiliations:** 1 Institute of Physics, National Academy of Sciences of Ukraine, pr. Nauky 46, Kyiv 03028, Ukraine; 2 Physics Department, University of Calabria, Ponte P. Bucci, Cubo 33B, 87036 Rende (CS), Italy; 3 CNR-NANOTEC, LiCryL and Centre of Excellence CEMIF. CAL, Ponte P. Bucci, Cubo 33B, 87036 Rende (CS), Italy; 4 Taras Shevchenko National University of Kyiv, Kyiv 01601, Ukraine

## Abstract

We report a strategy to assemble and manipulate nanoparticles arrays. The approach is based on the use of topological defects, namely disclination lines, created in chiral liquid crystals. The control of nanoparticle-loaded topological defects by low power light is demonstrated. Large-scale rotation, translation and deformation of quantum dots light-emitting chains is achieved by homogeneous LED illumination. Full reconfigurability and time stability make this approach attractive for future developments and applications.

Control of the spatial positioning of nanoparticles (NPs) over multiple length scales is very promising for new and more efficient nanotechnologies involving the field of material science, photonics, renewable energies, biomedical applications, and enabling lab-on-a-chip solutions (for sensing, imaging, drug delivering, etc.)^1^,^2^,^3^,^4^. At the same time it is a fertile ground for new fundamental science at the micro- and nano-scale. Different approaches^5^,^6^,^7^,^8^, either top-down and bottom-up, have been proposed to assemble NPs in a variety of environments suitable for controlling their interaction (electrostatic, magnetic, etc.). These enable to obtain highly ordered arrays of NPs exhibiting collective mechanical, photonic, electronic or magnetic properties with enormous impact^9^,^10^. Light is often good candidate for remote control and manipulation of microparticles^11^ and NPs^12^. Actual applications mainly involve optical tweezers^13^, whose control range is typically restricted to the micro-scale.

Exploiting new strategies to assemble 1D and 2D architectures of NPs and the ability to manipulate them is still a great challenge. In this sense, one can look for condensed matter structures with nanoscale dimensions as fine instruments for the task. A possible approach is the use of topological defects (TDs). They generally mark locations where disparate choices of a broken-symmetry in the system lead to irreconcilable differences^14^,^15^,^16^. The existence of TDs is a consequence of violations in the orientational order which occur in many soft materials (e.g. biological membranes, lipid monolayers, liquid crystals (LC))^17^,^18^,^19^. Defects in a nematic liquid crystal (NLC), as in any ordered medium, are places where the nematic order vanishes and the molecular director is thus ill defined. They occur in the form of isolated point or disclination line, when the NLC is subject to topological constraints. On the one hand, soft-matter based systems exhibit collective phenomena and high sensitivity to external stimuli. This permits to modify the orientational order even with moderate variations of the control parameters (temperature, electric, magnetic or optical fields, etc). Structures stabilized by TDs are extremely robust among various structures in soft matter, since the energy barrier required to destroy them is far beyond the thermal energy^20^. These localised distortions in order parameter can effectively trap nanoparticles^21^,^22^,^23^,^24^,^25^. The trapped particles further stabilize TDs, opening way for creation of the colloidaly stabilized defect networks in soft materials such as LC^26^,^27^,^28^. They behave as gels with a solid-like elasticity responsive to various external stimuli^28^,^29^. Trapping of particles by TDs was largely exploited for 2D and 3D assembling of colloids^20^,^29^,^30^,^31^. Moreover, the displacement of colloidal particles in the host environment by means of external fields (i.e. optical tweezers) affects the surrounding medium, including the TDs^31^. Thus, in the NPs-doped soft materials reciprocal advantages arise that convey towards a new approach to assemble and manipulate NPs.

The basic idea of the present study is to assemble and control NPs (namely quantum dots, QDs) by exploiting TDs induced in chiral NLC. Orientational TD lines (disclinations) appear between regions with different number of half-pitches of the cholesteric spiral^17^ or opposite chirality domains created in NLC by proper surface boundary conditions^31^,^32^. A small amount of a chiral dopant (ChD) in the NLC introduces an additional mesoscopic twist to the substrate-induced architectures (i.e. a twisting bias) and moves the disclination lines with respect to the configuration of a pure (achiral) NLC^33^. Here a photoresponsive azobenzene ChD, whose twisting power can be reversibly tuned by light, is used to control the position and shape of QDs-charged TDs. Confocal fluorescence microscopy shows that QDs are efficiently collected in the TD lines forming well-shaped fluorescing linear chains. Large rotations of cm-long QDs chains, μm-displacements of QDs lines array and curvature tuning of μm-long arch-like 2D QDs arrays are demonstrated. Moreover, a long time stability of the QDs structures and a good cyclic reproducibility of their manipulation is observed.

## Results and Discussion

Three kinds of LC cells, differing by anchoring conditions, have been investigated, which provide single, 1D and 2D arrays of TD lines. The empty cells have been assembled with asymmetric boundary conditions, various surface treatments and geometries have been exploited following mechanical and optical approaches as reported in *methods.* The cells were filled with a chiral NLC/QDs suspension, i.e. an eutectic NLC doped with 0.5 wt.% of photoresponsive left-handed ChD and 0.007 wt.% of CdSe/ZnS hydrophobic QDs (see *methods*). The ChD is an azo-dye containing chiral units, whose chemical formula is reported in reference^34^. It has two azo linkages, which undergo reversible *trans-cis* (*cis-trans*) isomerization under blue (green) light irradiation. In the *trans* configuration, ChD induces a cholesteric helix in the NLC with a pitch of 17.6 μm, which increases to 22.5 μm when the ChD is converted to the *cis* isomer.

**Figure 1:**
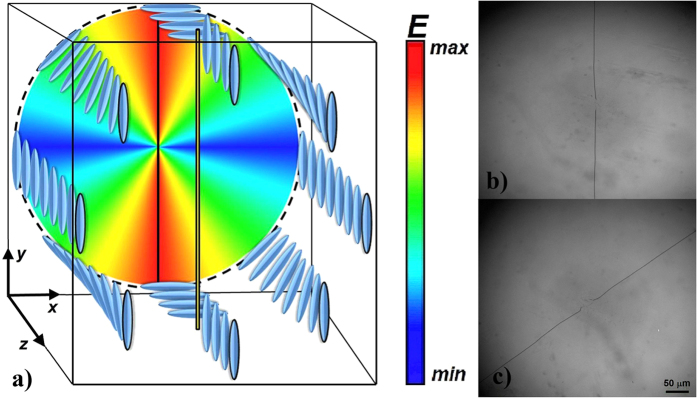
(**a**)Schematic representation of the molecular ordering in a *θ*-cell in the case of achiral liquid crystal. The colour code represents the Frank-Oseen free energy density of the medium. Unidirectional rubbing is parallel to *y* axis. The single disclination line occurs parallel to the homogeneous rubbing direction. (**b**) Optical microscope image of a 20 μm-thick *θ*-cell filled with QDs doped achiral NLC. (**c**) Optical microscope image of the same cell with the ChD. The addition of the ChD induces an intrinsic twist of the NLC and the disclination rotates by 2π + 0.9 rad. The images (**b,c**) were taken with a × 5 magnification objective. Scale mark indicates 50 μm.

The first cell, called *θ*-cell^35^, is manufactured from two anchoring substrates imposing unidirectional and circular planar alignment, respectively (see *methods*). When an achiral NLC is infiltrated between the substrates, opposite twist deformation of NLC is produced in the two halves of the cell 33. At the line where the left-handed (−π/2) and right-handed (π/2) twisted domains meet, the topological conflict arises and the disclination line appears^36^. This disclination line possesses topological strength^37^ *S* = ½, is parallel to the director on the surface with unidirectional alignment and is localized in the centre of the NLC thickness. A sketch of the director distribution in the *θ*-cell with the achiral NLC is presented in Fig.1a, and the micrograph of the *θ*-cell with the disclination is shown in Fig. 1b.

Addition of the ChD to the achiral NLC results in a spiral structure of the molecular director field, whose pitch is inversely proportional to the concentration of the dopant. The induced chirality forces the disclination to rotate from its initial position and the rotation sense is determined by the chirality sign of the dopant. The observed azimuthal rotation angle is *θ* = 2π(*L/p* − *n*), where *n* is the number of full rotations of the disclination line^33^, L is the thickness of the NLC film, and 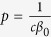 the pitch that depends on the ChD concentration *c* and its twisting power *β*_0_. The micrograph of the *θ*-cell with the chiral NLC is presented in Fig. 1c.

**Figure 2:**
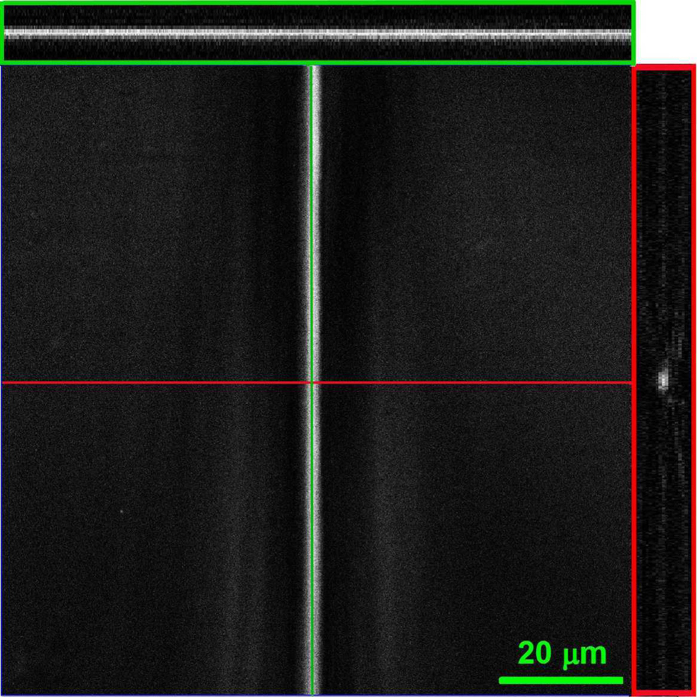
Confocal fluorescence microscope analysis of the TD line demonstrates QDs trapping. The longitudinal (upper green rectangle) and the transverse (right red rectangle) cross-sections show fluorescence from the QDs chain located in the centre of the cell with a cross section size of 1.5 ± 0.5 μm.

The localization of QDs in the chiral NLC cells is investigated by confocal fluorescence microscopy. Fig. 2 reports the 3D image stack, taken with the ×63 objective, of a 5 μm-thick *θ*-cell whose bottom substrate, in contact with the oil, is a 170 μm-thick glass coverslip. In order to ensure optimal sectioning of the sample along the z-axis, the pin-hole aperture has been set to achieve axial resolution of 0.4 μm. The top view as well as the longitudinal cross-section and the transverse cross-section of fluorescent pattern of the TD are reported in Fig. 2. The analysis confirms that the fluorescence is confined in a narrow line, with a transversal width of 1.5 ± 0.5 μm, and is uniform over the whole TD length (about 2 cm). The QDs line is located between the confining substrates, close to the middle of the NLC film.

**Figure 3:**
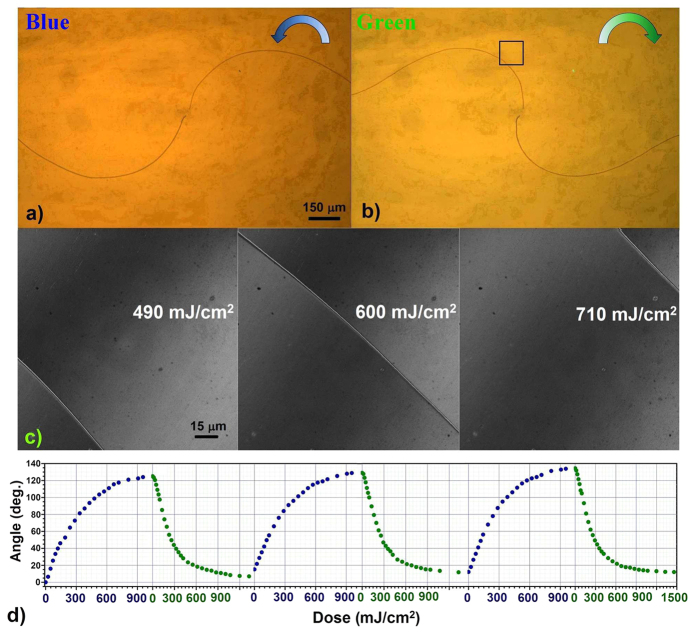
LED controlled reversible rotation of single NPs stripe. Starting from the initial position (shown in Fig. 1b), an anti-clockwise rotation of the TD line with trapped QDs occurs under blue LED illumination (**a**) and a subsequent clockwise rotation occurs under green LED irradiation (**b**) toward the initial position. The frames (**a,b**) correspond to a dose of 430 J/cm^2^ and 490 J/cm^2^ respectively. (**c**) A sequence of the confocal microscope images, referring to the black square region in (**b**), is shown for different green light dosages. The angle of rotation versus blue and green irradiation dosage is represented in (**d**) for three successive cycles, accounting for the full reconfigurability of the NPs line rotation.

When the *θ*-cell is irradiated with a blue LED (emission wavelength λ_blue_ = 400 nm), the light induces the *trans-cis* isomerization of the azobenzene ChD. Its twisting power under blue LED irradiation reduces to 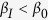 and also the rotation angle *θ* of the TD line decreases. Therefore, a continuous rotation of the TD line around the central singularity is expected, whose dependencies of direction and rate on the light dose are determined by the ChD handedness and light intensity, respectively^32^. Subsequent irradiation with a green LED (emission wavelength λ_*green*_ = 560 nm) activates the reverse *cis-trans* isomerization of the ChD, increasing its twisting power up to *β*_0_ and then inducing a rotation of the TD line towards the initial position. It is worth noting that the spontaneous *cis-trans* isomerization, which recovers the initial configuration after blue LED irradiation, takes several hours. The video 1 (see supplementary information) shows the counter-clockwise rotation of the QDs-rich TD line in the *θ*-cell under blue LED illumination and the following clockwise rotation resulting by the green LED irradiation. The video has been shoot under uniform light intensity irradiation of the *θ*-cell, for both the blue and green LEDs, and is speeded up by 8 times. Figure 3a shows the frame of the video 1, taken during the blue LED irradiation, which corresponds to a blue light dose of 430 mJ/cm^2^. The rotation angle of the TD line measured near the central singularity is approximately π/2. The frame of the same video 1 reported in Fig. 3b shows the clockwise rotation of the TD line towards the initial position. It refers to the green LED irradiation at a dose of 490 mJ/cm^2^, consecutive to a blue LED irradiation with a dose of 950 mJ/cm^2^.

Due to the uniform LED light illumination, the instantaneous concentration of the ChD isomers is uniform over the whole cell and reaches its steady state typically faster than the rearrangement of the molecular director. Therefore, the reorientation dynamic of the TD line is determined by the elastic-viscous properties of the LC. The linear distance traveled by the TD line on its outer ends is larger than the distance traveled closer to the central singularity. Because of this, the inner TD edge always leads the rotation with respect to the outer edges, producing a left-handed (right-hand) spiral during the counter-clockwise (clockwise) rotation observed under blue (green) LED illumination (see Fig. 3a,b).

The confocal fluorescence images in the sequence of Fig. 3c refer to the black square area in Fig. 3b, and have been taken with the x20 objective during the green LED irradiation. The sequence makes evidence of the displacement of the QDs-rich TD line at different doses. Large rotation (hundred of degrees) and displacement (centimetres) of the cm-long QDs chain have been obtained, preserving both TD line stability and QDs trapping. In addition, the complete reversibility of the movement of the QDs-rich TD line demonstrate a good fatigue resistance. This is evident in Fig. 3d where three subsequent blue-green irradiation cycles are shown.

The second and third cells, generating 1D and 2D periodic patterns of QDs lines, are assembled using one unidirectional orienting substrate and a second substrate covered with a polarization-sensitive azo-dye film^38^. Exploiting polarization holographic recording in combination with the photoaligning command substrates, the opposite-sign twist configuration is realized periodically, thus providing the condition for properly designed TD arrays.

**Figure 4:**
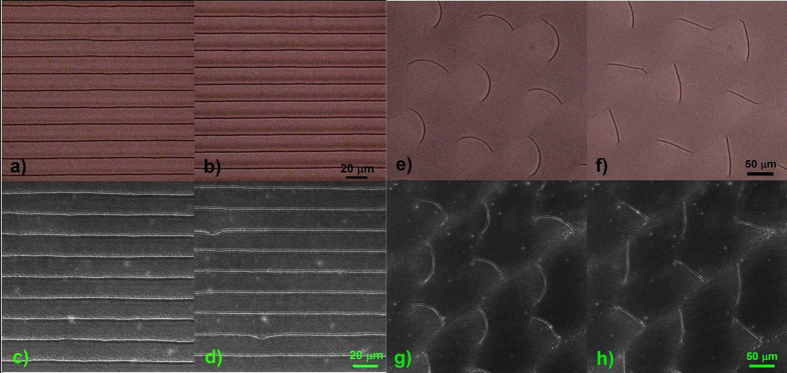
LED controlled displacement and curving of NP arrays, 1D (linear) and 2D (arch-like). Bright-field (**a,b**) and confocal (**c,d**) microscope images are taken for 1D array of TD lines which appear in QDs-doped chiral LC confined between an uniform planar and a periodic planar aligning substrates. The array has a spatial periodicity of 16 μm and after a blue light irradiation dose of 20 J/cm^2^ undergoes a displacement on 5 μm perpendicular to the TDs lines (**b,d**). The comparative analyses of the bright field and confocal microscope images confirm the trapping of QDs within the TD cores and their stability during the displacement. Bright-field (**e,f**) and confocal (**g,h**) microscope images are taken for 2D arrays of TD arch-like lines. The array has a spatial periodicity of 150 μm and exhibits an arch-like structure with different orientation but uniform curvature, with a radius of 40 ± 10 μm (**e,g**) which relies on the LC chirality (i.e. ChD concentration). After a blue light irradiation with dose of 20 J/cm^2^ the radius of curvature increases to 250 ± 10 μm (**f,h**). The comparative analyses of the bright field and confocal microscope images confirm the trapping of QDs within the TD regions and their stability during the deformation.

Two coherent Gaussian beams of equal intensities and orthogonal circular polarizations are used to record the hologram on the photosensitive substrate, giving a continuously rotating linear polarization pattern. 1D periodic parallel QDs-rich TD lines of the same kind described before (singular core, *S* = 1/2) are produced with a spatial periodicity of 16 µm. Figure 4a,b show the bright-field microscope images, before and after the blue LED illumination of the chiral NLC/QDs layer confined between an unidirectional planar aligning substrate and a periodic planar photo-aligning substrate, providing periodically modulated alignment direction^38^(see video 2 in the supplementary information). The corresponding confocal fluorescence microscope images are represented in Fig. 4c,d, respectively. The blue LED illumination induces a parallel displacement of the QDs stripes up to 5 μm for a dose of 20 mJ/cm^2^, while the subsequent green light illumination (for the same dose) recovers their initial position.

A polarization hologram recorded by two pairs of laser beams on the photoaligning substrate is exploited to obtain the 2D pattern of TD lines^39^. The four beams of equal intensities having perpendicular planes of incidence and orthogonal circular polarizations, irradiate the photosensitive film producing a 2D polarization pattern with spatial periodicity of 150 μm. The corresponding TD lines in the chiral NLC/QDs suspension are shown in Fig. 4e,f. The TD lines, 50 ± 5 μm long, occur between two defects points (see Fig. 4e) whose position is determined only by the spatially modulated anchoring conditions imposed by the boundary substrates. On the other hand, the arch-like configuration of the TD lines structure, with a radius of curvature of 40 ± 10 μm, is strictly related to the LC chirality (see also video 3 in the supplementary information). In Fig. 4g the confocal fluorescence microscope image proves that QDs are trapped by the arch-shaped TD lines, whose fluorescence width is about 1 μm. As a consequence of the blue LED illumination, the QDs-rich TD lines undergo an evident distortion from arch-shaped lines towards straight lines, by increasing the curvature radius consistently (up to 250 μm) as shown in Fig. 4h. In both cases the NPs are always trapped in the core of the disclinations and move with them as reported in Fig. 3c.

To conclude, we report on a methodological approach to assemble and to displace NPs over large scales. The strategy is based on the ability of TD structures that appear in chiral soft material to trap NPs. Then, we exploit the capabilities to manipulate TDs position from the micrometre to the centimetre scale via cooperative phenomena guided by a photochemical process which can be remotely controlled by simple incoherent illumination. In particular, we demonstrate QDs assembling in defect lines created in the cells filled with NLC/QDs suspension with phototunable chirality. Single, 1D and 2D lines arrays of QDs, whose length ranges from the micrometer to centimetre, have been created. Control of their position and shape has been also explored by low-power LED illumination. Large distance displacement, rotation and changes of the lines curvature have been demonstrated. The reversibility of the involved photochemical processes enables complete reconfiguration of the NP structures.

We believe that the proposed approach may lead to design a variety of large-scale NPs geometries with tunable collective properties, offering new opportunities for the development of feasible and switchable macroscale nanotechnology and nanomaterials. In particular, it will be possible to construct tunable emitters or metamaterials by embedding quantum dots in the orientational defects. Additional perspectives may arise by immobilising the light-induced nanostructure in a polymer LC network or by removing the LC in order to get a pure superstructure of NPs^40^. This option further extends the application possibilities of the proposed approach.

## Methods

### Materials

The polarization sensitive dye film used on glass substrates to record polarization holograms has been prepared from a solution of the azo-dye Brilliant Yellow (Sigma-Aldrich) in dimethyl formamide (1% by weight) by spin coating at 3000 rpm for 60 s. The coated glass substrates were baked at 90 °C for one hour in order to let the solvent evaporate before to be used for assembling the cell.

The chiral photoresponsive dopant, ChD, used to induce the mesoscopic twist is an azo-dye containing chiral units, whose chemical formula is reported in reference^34^. It has two azo linkages, which can lead to reversible *trans–cis* isomerization of azo configurations under light irradiation. Blue light at 400 nm induces *trans-cis* process, while green light recovers *trans* configurations. The ChD induced a cholesteric spiral in a LC E7 (Merck) with the pitch 22.7 μm at the concentration of the ChD 0.5% by weight.

The quantum dots used are CdSe QDs (diameter 2.5 nm) with a shell of ZnS (0.6 nm) from PlasmaChem GmbH. They absorb in the blue region of the electromagnetic spectrum and have the maximum emission at (530 ±5) nm. The QDs were dissolved in chloroform (0.04% by weight) under stirring and then the chiral LC mixture (E7, by Merck, plus ChD) was added to the solution. After chloroform evaporation, a suspension with a concentration of 0.007% in weight of QDs in LC was obtained. The suspension was infiltrated in the cells at the isotropic phase and subsequently cooled down in the LC phase.

### Devices preparation

Three different kinds of devices have been investigated. The first one, generating a single TD line, was obtained using two glass substrates covered by a 20 nm-thick polyimide film (measured by X-ray reflectometry) which provides planar anchoring of the LC molecules on the substrate. The rubbing process was performed mechanically, unidirectional for one substrate using a velvet-wrapped roller and circular for the second one using a rotating disk. The spacers between glasses provided a cell thickness of 20 μm, while the transversal dimensions of the cell are 2 cm × 3 cm. The second and third devices, generating 1D and 2D arrays of TD lines, have been obtained using one unidirectional orienting substrate and a second substrate with a tens of nm thick polarization sensitive azo-dye film able to provide holograms-designed anchoring condition^38^. The photo-aligning patterns have been generated exploiting polarization holographic recording with two different schemes. The 1D hologram was recorded on the substrate covered by the photosensitive film by exposing it for 120 s to the interference of two coherent Gaussian beams (Ar^+^ laser, wavelength λ = 458 nm) of equal intensities (15 mW/cm^2^) and orthogonal circular polarizations. The resulting 1D polarization pattern is a continuously rotating linear polarization with a spatial periodicity of 16 μm, depending on the crossing angle of the recording beams, which provides a periodic planar alignment of the NLC^34^.

The 2D hologram was recorded exposing the photoaligning glass substrate to the interference of two pairs of beams with perpendicular planes of incidence and orthogonal circular polarizations, using the optical system reported in reference^35^. The intensity of each beam was 10 mW/cm^2^ while a phase shift of π∕2 between consecutive beams suppresses amplitude modulations of the optical field in the superposition region. The resulting 2D polarization pattern is reported in reference^35^, the spatial periodicity of the 2D pattern was 150 μm depending on the crossing angle of the recording beams.

In both cases the cell thickness was 6 μm and the area of the hologram was about 1 cm^2^.

### Imaging techniques

Bright-field optical microscopy investigations were performed at × 5 and × 20 on a Zeiss AxioScope upright microscope. Confocal images were collected with a Zeiss LSM 720 inverted laser scanning microscope using a × 20 (NA = 0.9) dry or a × 63 (NA = 1.4) oil immersion objective and 458 nm Ar^+^ laser excitation. All fluorescence images were acquired in the wavelength range 530 ± 30 nm, peaked at the nominal emission of the QDs, at 1024 × 1024 pixel resolution in the xy plane, by averaging a sequence of 8 acquisitions. 2D images were taken with the × 20 objective, while the 3D image stack was taken with the × 63 objective on a 5 μm-thick sample whose bottom substrate, in contact with the oil, is a 170 μm-thick glass coverslip. In order to ensure optimal sectioning of the sample along the z-axis, the pin-hole aperture was set to achieve axial resolution of 0.4 μm.

## Additional Information

**How to cite this article**: Kasyanyuk, D. *et al.* Light manipulation of nanoparticles in arrays of topological defects. *Sci. Rep.* **6**, 20742; doi: 10.1038/srep20742 (2016).

## Supplementary Material

Supplementary Movie 1

Supplementary Movie 2

Supplementary Movie 3
